# Alternating Consecutive Maximum Contraction as a Test of Muscle Function in Athletes Following ACL Reconstruction

**DOI:** 10.2478/v10078-012-0074-9

**Published:** 2012-12-30

**Authors:** Olivera M. Knezevic, Dragan M. Mirkov, Marko Kadija, Darko Milovanovic, Slobodan Jaric

**Affiliations:** 1 University of Belgrade, Faculty of Sport and Physical Education, Belgrade, Serbia.; 2 University of Belgrade, Institute for Medical Research, Belgrade, Serbia.; 3 Clinical Centre of Serbia, Institute for Orthopaedic Surgery and Traumatology, Belgrade, Serbia.; 4 University of Delaware, Department of Kinesiology and Applied Physiology, Newark, DE, USA. .

**Keywords:** Strength, Knee, flexor, extensor, Rehabilitation

## Abstract

The novel test based on isometric alternating consecutive maximal contractions performed by two antagonistic muscles has been recently proposed as a test of muscle function in healthy subjects. The aim of this study was to evaluate reliability and sensitivity of a novel test as a test of knee muscles function in athletes recovering from anterior cruciate ligament reconstruction. Fifteen male athletes with recent ligament reconstruction (4.0 ± 0.1 months following the surgery) and 15 sport and physical education students participated in the study. Peak torques of the quadriceps and hamstring muscles assessed both through the alternating consecutive maximal contractions and standard isokinetic test performed at 60 º/s and 180 º/s served for calculation of the hamstrings-to-quadriceps ratio and the bilateral difference in strength. When applied on individuals recovering from anterior cruciate ligament reconstruction, the novel test revealed a high within-day reliability and sensitivity for detecting imbalances both between antagonistic and between contralateral muscles. The present findings suggest that alternating consecutive maximal contractions could be used as a test of muscle function that is either complementary or alternative to the isokinetic test, particularly in the laboratories where the isokinetic devices are not available. Potential advantages of the novel test could be both a brief testing procedure and a possibility to conduct it using relatively inexpensive devices such as custom made kits containing a single one-axis force transducer.

## Introduction

The Anterior Cruciate Ligament (ACL) injury is one of the most frequent injuries associated with athletic activity (Kvist et al., 2001). The quadriceps strength has been shown to have a strong relationship with a positive outcome regarding the indices of recovery following the ACL reconstruction (Kannus, 1988; Hiemstra et al., 2004; Lautamies et al., 2008; Hartigan et al., 2011). The change in muscle function over the course of rehabilitation has been usually assessed by standard isokinetic tests (IKT) (Ostenberg et al., 1998; Keays et al., 2001; Kvist et al., 2001).These protocols have been mainly designed to evaluate the maximal torque production of the involved/uninvolved side following either an ACL injury or reconstruction (Ostenberg et al., 1998; Keays et al., 2001). Specifically, IKT has been usually applied to quantify the side-to-side difference and strength balance between the hamstrings and quadriceps (Moisala et al., 2007; Myer et al., 2008; Kadija et al., 2010). In particular, the side-to-side difference has been usually expressed as the bilateral difference in strength (BLD), representing the percent difference between the strength test outcomes of the contralateral limbs. In addition, the strength imbalance ratio known as the hamstrings-toquadriceps ratio (HQ ratio) has been used to assess the balance between two antagonistic muscles that could be important for knee joint stabilization (Coombs and Garbutt, 2002; Holcomb et al., 2007; Moisala et al., 2007).

Even though IKT could be generally considered as a standard method for the assessment of muscle function after ACL reconstruction, the use of isometric test could also be considered, particularly when isokinetic dynamometers are not available (Jaric, 2002). Therefore, a novel test based on isometric alternating consecutive maximal contractions (ACMC) has been recently proposed as either an alternative or complementary test of muscle function to IKT (Suzovic et al., 2008; Bozic et al., 2011; Bozic et al., 2012). The ACMC test is typically performed by two antagonistic muscles (i.e., quadriceps and hamstrings) and is based on isometric muscle contractions performed in alternating manner. The main rationale for the initial selection of this test is its general correspondence with the regime of muscular contraction typical for rapid and cyclic movement, rather than with long lasting maximum force exertions that are typical for standard isometric and IKT tests (Suzovic et al., 2008). When compared with a standard isometric test, ACMC could also have important methodological advantages, such as being based on a simple and fatigue-free procedure for testing two antagonistic muscles within a single trial, it exposes the muscle and joint tissues to relatively low and transient forces (Bozic et al., 2011).

The ACMC test has been so far evaluated only on healthy subjects and the obtained results revealed high reliability, moderate-to-high concurrent and external validity, and a sensitivity that allows for discerning among the individuals of different athletic training and physical activity history (Suzovic et al., 2008; Bozic et al., 2012). However, to be applied on clinical populations, ACMC could need both a further evaluation and some specific methodological adjustments. Furthermore, the outcome of ACMC should be compared with the standard IKT, which is routinely used in the assessment of muscle function in individuals recovering after ACL reconstruction (ACLR).

Therefore, the main aim of the present study was to evaluate ACMC as a test of muscle function in ACLR individuals. The specific aims were to evaluate the within-day reliability of the novel ACMC test when used to assess the muscle function in ACLR individuals, and to explore the sensitivity of ACMC for detecting the asymmetries in strength both between contralateral limbs and between antagonistic muscle groups, and to compare it between ACLR and healthy individuals. Regarding the expected outcome, we hypothesized that the ACMC test would be a reliable and sensitive tool for the assessment of muscle function in the patients following the ACLR. The results were expected to motivate further development of ACMC as a routine test of muscle function in ACLR individuals and, possibly, other clinical populations.

## Material and Methods

### Participants

Two groups of subjects were recruited for the purpose of this study. For the assessment of reliability and concurrent validity of the ACMC test, we tested 15 male soccer, handball and judo competitors (ACLR group; age 22 ± 4 years; body mass 84.0 ± 11.1 kg; body height 180.3 ± 4.0 cm) with recent ACL reconstruction (4.0 ± 0.1 months following the surgery). The inclusion criteria for the ACLR group were: first ACL injury, no other knee ligaments injured, and no history of concurrent fractures, osteoarthritis, or hereditary and neuromuscular diseases. The ACL reconstruction procedure was identical in all patients performed by the same surgeon, using the bone-patellar-bone tendon (BPTB) autograft. BPTB was a surgeon’s graft of choice at the time when this study was conducted. Following the surgery, the patients were allocated to a standard postoperative rehabilitation program for athletes. They were included in the study only after being discharged from physical therapy and cleared from the surgeon to return to sport activities.

Additionally, for the assessment of the sensitivity of the ACMC, 15 healthy and male physical education students were also tested (Control group; age 23 ± 2 years; body mass 80.8 ± 8.5 kg; body height 181.6 ± 6.5 cm). These individuals were active through their standard academic curriculum (on average 12 activity classes per week that mainly included high intensity exercises with inclusion of strength training). None of the participants selected for the control group suffered from neurological disease or recent injuries that could compromise the tested outcomes.

All participants received a complete explanation regarding the purpose and procedures of the study, as well as possible risks. Ethical clearance for the study was obtained from the IRB committees of both the relevant hospital and university. In line with Helsinki Declaration, the subjects signed the institutionally approved informed consents.

### Procedures

All measurements were performed within a university research laboratory, using a Kin-Com isokinetic dynamometer (Chatex Corp., Chattanooga, TN). Prior to the muscle strength testing each subject was given a five minute warm-up period on a stationary bicycle, followed by passive stretching exercises. Thereafter, the subject was fixed to the testing apparatus with the tight straps used to fix his pelvis, thigh, and malleoli, while tightly holding the sides of the dynamometer chair. The axis of the knee was aligned with the axis of rotation of the dynamometer. The same test leader supervised all tests. A detailed explanation and qualified demonstration were provided prior to each muscle function test and a standardized verbal encouragement was used. A real-time feedback regarding the current force was shown at a display positioned in front of the subject.

The muscle function of hamstrings and quadriceps of both legs was tested in the ACLR group (both the uninvolved and involved leg), and in the control group (dominant and non-dominant leg; dominant leg was defined as the preferred kicking leg) through both IKT and ACMC. The muscle function tests (i.e., isokinetic and ACMC) were performed in random order on two separate days, with 48 hours rest between them.

### Measurements

IKT was conducted according to the most frequently applied procedures for quadriceps and hamstrings strength assessment (Kannus, 1988; Moisala et al., 2007; Lautamies et al., 2008). The range of motion was set to 80° (i.e., from 10° to 90° of knee flexion). In particular, five maximum consecutive concentric contractions of two muscles were separately performed at 60 º/s (IKT60) and 180 º/s (IKT180) that represent the most often applied angular velocities in the literature (Kannus, 1988; Dvir, 1995; Moisala et al., 2007; Lautamies et al., 2008). One familiarization trial was performed prior to testing, which included five submaximal repetitions. Thereafter, two trials were performed at each angular velocity with one minute rest between them, while the rest between tests conducted at different velocities was two minutes (see Jaric 2002 for review of similar procedures).

Since it is well known that muscle strength depends on the knee angle, applying ACMC at various angles would result in different HQ ratios. A pilot testing was conducted prior to the present experiment where the isometric strength of quadriceps and hamstring muscles was tested through the range of knee angles (100, 120, 140 and 160 degrees) and the results revealed that each muscle exerts the same percent of their maximum isometric torque at the knee angle of 45º. As a result, the same angle was selected for testing ACMC.

The protocol previously described by Bozic et al. (2012) was applied in ACMC testing. In short, the participants were instructed “to consecutively exert the alternating maximum contractions of quadriceps and hamstrings as strong and as quickly as possible” and therefore, the ACMC frequency could be considered as self-selected. The trial duration covered at least five full periods of ACMC force. The familiarization procedure conducted prior to testing included five submaximal repetitions. Thereafter, two trials were performed with one-minute rest between them. The uninvolved (ACLR group) and the dominant leg (Control group) were always tested first. None of the patients reported a pain in the involved leg prior to and during the testing.

A custom made LabVIEW application (National Instruments Corp. Austin, TX, USA) was used for data acquisition and processing of the ACMC and IKT. The force-time curves were recorded at a sample rate of 500 Hz and low-pass filtered (10 Hz) using a fourth-order (zero-phase lag) Butterworth filter (Bozic et al., 2011; 2012).

**Figure 1:**
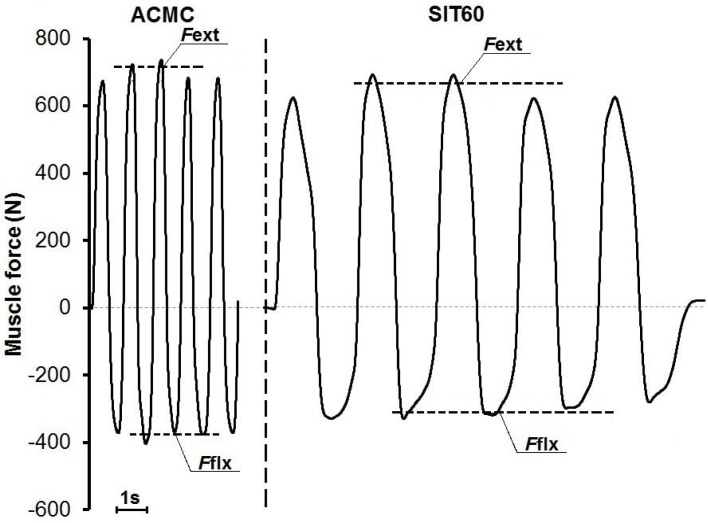
Typical force-time curves observed in ACMC and a standard isokinetic test at 60º/s IKT60 = isokinetic test at 60º/s; ACMC = alternating consecutive maximum contractions

The force maxima provided the peak forces that were multiplied by the length of individual lever arms to calculate the peak torques (PT) for both the quadriceps and hamstring muscles. Since all dependent variables were based on the calculated muscle torques, normalization for body size was not needed (Jaric et al., 2002). The self-selected frequency of ACMC was calculated from the time intervals between the consecutive force peaks. All variables recorded in IKT and ACMC tests were calculated as average values obtained from the middle three periods of the individual trials. More specifically, the peak values observed from the middle three cycles of IKT and ACMC force profiles provided averaged PT of quadriceps (PT_Q_) and hamstrings (PT_H_; Figure 1). The same LabVIEW application was used to provide real-time force profiles for visual inspection.

PT_Q_ and PT_H_ observed from both ACMC and IKT performed at two angular velocities served for calculation of HQ ratios as:

eq.1 $$HQ_{\mathrm{ratio}}=\frac{PT_{H}}{PT_{Q}}$$

Bilateral difference (BLD) in strength between the involved and uninvolved leg, as well as between the non-dominant and dominant leg was calculated as (Moisala et al., 2007):

$$BLD=\frac{PT_{\mathrm{uninvolved}}-PT_{\mathrm{involved}}}{PT_{\mathrm{uninvolved}}}\times 100$$

eq.2 $$BLD=\frac{PT_{\mathrm{dominant}}-PT_{\mathrm{non-dominant}}}{PT_{\mathrm{dominant}}}\times 100$$

In both experiments the trials that revealed higher PT (in IKT) or sum of PT_Q_ and PT_H_ (in ACMC) were taken for further analysis.

### Statistical analysis

To assess the within-session reliability, the intra-class correlation coefficients (ICC) of the repeated trials were calculated as a measure of relative reliability or consistency of measurement (Hopkins, 2000). The coefficient of variation (CV) was used as a measure of absolute (i.e., within-individual) reliability (Hopkins, 2000), while the paired t-test was applied to detect the systematic bias among two consecutive trials. The mixed ANOVA design (4 x 3) was used to identify significant differences across HQ ratios (factors: leg and test), and across BLD (2 x 4 x 3 [factors: group, muscle and test]). When significant main effects were found, where it was needed, the Bonferroni post-hoc test was performed. The level of statistical significance was set at *p* = 0.05. Data were analyzed using the SPSS 17.0 software (SPSS Inc. Chicago, IL, USA).

## Results

The indices of the within-day reliability of *PT* were exceptionally high in all tests (median ICC = 0.97), while the within-subject (i.e., absolute) reliability expressed as CV varied from 2.9 % to 10.3 % (Table 1). There were no significant differences among the consecutive trials (t-test values ranged from −1.98 to 1.7; all *p* > 0.05).

**Figure 2:**
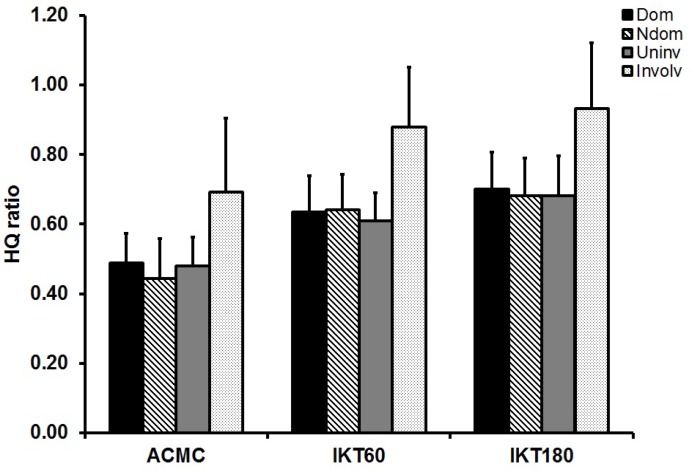
HQ ratios obtained from ACMC and isokinetic tests (the individual bars represent mean [SD]) Dom = dominant leg; Ndom = non-dominant leg; Uninv = uninvolved leg; Involv = involved leg; ACMC = alternating consecutive maximum contractions; IKT60 = isokinetic test at 60º/s; IKT180 = isokinetic test at 180º/s

The significant main effect of the leg (F_3.51_ = 17.03; *p* < 0.01) and the test (F_2.102_ = 102.54; *p* < 0.01), but no interaction between them (F_3.51_ = .71; *p* > 0.05) was found when HQ ratios of IKT and the ACMC test were compared (Figure 2). Regarding the differences between the legs, the Bonferroni *post hoc* test revealed higher *HQ ratio* of the involved leg than either of the uninvolved (*p* < 0.01), or dominant (*p* < 0.01) or non-dominant leg (*p* < 0.01), while HQ ratios of the uninvolved leg were not different from HQ ratios of the dominant and non-dominant leg. Regarding the main effect of the test, HQ ratios obtained from ACMC were lower than those obtained from IKT60 (*p* < 0.01) or IKT180 (*p* < 0.01). Finally, HQ ratios obtained from IKT60 were lower than those obtained from IKT180 (*p* < 0.05).

**Figure 3:**
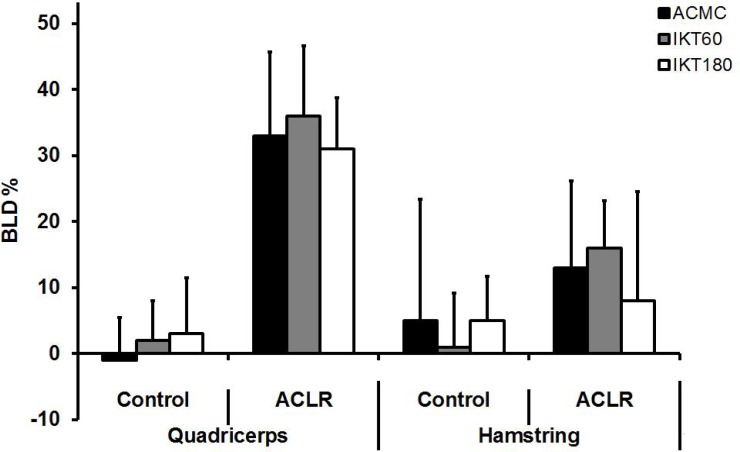
Bilateral difference (BLD) in strength of quadriceps and hamstring muscles between contralateral limbs obtained from ACMC and isokinetic tests; mean (SD)

Regarding the BLD (Figure 3), the results revealed significant main effect of the group (F_1,47_ = 97.709; p < 0.01), muscle (F_1,47_ = 17.789; p < 0.01), as well as the interactions between the group and test (F_2,164_ = 4.050; *p* < 0.05) and between the group and muscle (F_1,47_ = 23.877; *p* < .01). In particular, BLD was higher in quadriceps than in hamstring mainly due to the higher values obtained from ALCR than from the Control group. Regardless of the applied test, the highest BLD was found in the quadriceps strength recorded from the ACLR group (*p* < 0.01).

## Discussion

The aim of the present study was to evaluate the isometric alternating consecutive maximal contraction (ACMC) as a test of muscle function in patients following the ACL reconstruction and to compare it with the standard isokinetic test (IKT). In general, we found the reliability and sensitivity of ACMC to be similar to IKT.

Regarding the reliability, most of the recorded PT obtained from ACMC and IKT revealed high test-retest correlations, low within-subject variations, and no bias between consecutive trials. Somewhat higher CV associated with ACMC variables observed in the involved leg could be partly a consequence of differences in the rehabilitation progress across the participants. The observed indices of reliability are generally similar to those observed from ACMC applied on healthy individuals (Bozic et al., 2011; 2012) or even higher than the values obtained from previous evaluations of IKT (Harding et al., 1988; Montgomery et al., 1989; Impellizzeri et al., 2008; see also Jaric 2002 for review). Overall, the data suggest that the ACMC could be routinely applied after very few familiarization trials, while still being able to detect either the relative differences or effects below 10 %.

The current study also provides indices of sensitivity of ACMC regarding the differences in muscle function among the two tested groups. Note that both HQ ratios and BLD calculated from ACMC proved to be sensitive enough to detect the effects of ACL reconstruction on muscle strength typically recorded by IKT. These findings are in line with previous studies suggesting that both the within leg muscle imbalance (i.e., HQ ratio), and between muscle differences (BLD) typical for ACLR individuals mainly originate from a relatively impaired quadriceps muscle strength due to impact from donor site morbidity from BPTB graft harvesting (Figure 3) (Keays et al., 2001; Anderson et al., 2002; Kobayashi et al., 2004; Ageberg et al., 2008; Pua et al., 2008).

Despite the demonstrated similarities in the properties of ACMC and IKT regarding their reliability and sensitivity, ACMC could also have some important properties that could be considered as a potential advantage of ACMC over IKT. First, although ACMC does not allow for utilization of the stretch shortening cycle due to its static conditions, the consecutive exertion of maximum forces of two antagonistic muscles could allow for action of a number neural networks and mechanisms generally believed to contribute in muscle excitation in cyclic movements (e.g., central pattern generator, or reciprocal inhibition (Latash, 2008)). Therefore, one could presume that the potential similarity of ACMC force pattern with a number of functional motor tasks speak in favor of ACMC’s face validity at least with respect to rapid reversal and cyclic movements. Second, ACMC could be based on an exceptionally brief procedure for testing two antagonistic muscles than IKT, and also could require very few practice trials, if any. Third, although the individual PT was comparable in two tests, those exerted in ACMC reveal shorter duration (Figure 1). That could not only resolve the problem of both the muscle and mental fatigue associated with multiple velocities testing protocols (Zemach et al., 2009) that have been considered as a potential limitation of IKT (particularly when performed at lower angular velocities) (Dvir, 1995; Zemach et al., 2009), but also decrease the overall loading of the muscle and joint connective tissues. Finally, although all tests within the present study were performed on an isokinetic device, note that ACMC could be performed on a much simpler and cheaper devices, including custom made kits containing a single one-axis force transducer (Suzovic et al., 2008; Bozic et al., 2011; 2012).

The limitations of the present study could originate from several factors. The simple size was relatively limited not only in number, but also in graft choice (only patients with BPTB graft were included), while the population homogeneity could be questioned since the ACLR participants were recruited from different sport disciplines. Additionally, the lack of female subject could also be a limiting factor, considering the higher incidence of ACL injuries in female athletes (Myer et al., 2008; 2009). Due to its cross-sectional design, the present study did not allow for controlling the rehabilitation progress that could have had some confounding effects on the observed outcomes.

To summarize, when applied on ACLR patients, the ACMC test revealed a high within-day reliability as well an adequate sensitivity for discerning both between the legs and between the muscles. Although the properties of the evaluated novel test proved to be comparable with the routinely applied IKT, ACMC could still have some important methodological advantages. Among them could be a briefer and fatigue-free procedure for testing two antagonistic muscles. In addition, ACMC could be performed on a simple and cheap device, including custom made kits containing a single one-axis force transducer (Suzovic et al., 2008). Therefore, the present findings generally suggest that ACMC could be developed as a test of muscle function following the ACL reconstruction, and that it could be either complementary or alternative to IKT, particularly in cases when isokinetic devices are not available.

## Figures and Tables

**Table 1:** Within-day reliability of peak torques (N·m) obtained from two muscle function tests in the ACLR group

	Trial 1 Mean ± SD	Trial 2 Mean ± SD	Change in mean %	T value	CV %	ICC	95% CI

*Quadriceps (uninvolved leg)*							
IKT60	172.8 ± 35.7	175.5 ± 37.8	1.6	−0.8	4.7	0.97	0.92 – 0.98
IKT180	128.4 ± 27.0	125.7 ± 25.2	−2.1	1.7	2.9	0.97	0.95 – 0.99
ACMC	192.0 ± 44.9	195.4 ± 48.5	1.8	−1.55	3.3	0.99	0.97 – 0.99
*Hamstrings (uninvolved leg)*							
IKT60	100.1 ± 25.5	101.2 ± 21.1	0.1	−0.1	5.1	0.95	0.87 – 0.98
IKT180	83.9 ± 19.5	82.4 ± 16.8	−1.8	0.92	4.2	0.97	0.92 – 0.99
ACMC	91.3 ± 25.7	92.1 ± 29.7	0.9	−0.37	5.7	0.96	0.90 – 0.98
*Quadriceps (involved leg)*							
IKT60	103.5 ± 40.1	109.1 ± 34.4	5.4	−1.87	8.3	0.95	0.86 – 0.98
IKT180	88.0 ± 23.1	89.6 ± 24.6	1.8	−1.57	2.9	0.99	0.98 – 1.00
ACMC	112.7 ± 40.1	119.4 ± 41.9	5.9	−1.98	9.2	0.95	0.88 – 0.98
*Hamstrings (involved leg)*							
IKT60	94.8 ± 31.0	94.4 ± 29.3	−0.4	0.34	3.4	0.99	0.95 – 1.00
IKT180	76.9 ± 20.1	77.6 ± 21.7	0.9	−0.66	3.3	0.99	0.97 – 1.00
ACMC	71.4 ± 22.3	77.3 ± 28.7	8.3	−1.74	10.3	0.89	0.75 – 0.96

CV = coefficient of variation; ICC = intra-class correlation coefficient; CI 95 % = confidence interval; IKT = isokinetic test performed at 60 º/s and 180 º/s; ACMC = alternating consecutive maximal contractions
